# Amino acid enhanced phosphorus fertilizers enhance wheat yield and efficiency by optimizing soil phosphorus pools in wheat fields for soil phosphorus risk management

**DOI:** 10.3389/fpls.2026.1910255

**Published:** 2026-08-06

**Authors:** Lin Cheng, Linchuan Zhan, Zongya Wang, Ximei Cao, Tingting Xu, Hongyan Wu

**Affiliations:** 1 Department of Hydraulic Engineering, Wanjiang University of Technology, Maanshan, Anhui, China; 2 Anhui Provincial Key Laboratory of Nutrient Cycling and Arable Land Conservation, Soil and Fertilizer Institute, Anhui Academy of Agricultural Sciences, Hefei, Anhui, China

**Keywords:** amino acid enhanced phosphorus fertilizer, phosphorus fractions, phosphorus use efficiency, soil phosphorus pool, wheat

## Abstract

Developing amino acid-based biostimulant-enhanced phosphate fertilizers can help improve fertilizer use efficiency and reduce environmental emissions. However, under the background of phosphorus (P) risk management, the mechanism by which amino acid-enhanced phosphate fertilizers affect the transformation characteristics of soil P remains unclear. This study aimed to explore the effects of amino acid addition on soil P pools, crop yield, and phosphate fertilizer utilization through a soil column experiment. Five treatments were set up: (1) no fertilizer control (CK), (2) no phosphate fertilizer control (NK), (3) application of diammonium phosphate (P), (4) application of 0.5% amino acid-enriched diammonium phosphate (AP0.5), and (5) application of 5% amino acid-enriched diammonium phosphate (AP5), with six replicates for each treatment. The results showed that amino acid-enriched phosphate fertilizers promoted the absorption and utilization of P by wheat, and improved the yield and PUE, with the most significant effect in the AP0.5 treatment. Compared with the P treatment, the soil Olsen-P and Labile-P in the AP0.5 and AP5 treatments increased by 29.76% - 33.45% and 4.49% - 5.11%, respectively, with significant differences. However, there were no significant differences in the above indicators among treatments with different amino acid addition amounts. Path analysis between different P fractions and Olsen-P showed that H_2_O-P, NaHCO_3_-P_i_ and NaOH-P_i_ had the greatest direct effects on Olsen-P; while NaHCO_3_-Po and NaOH-Po may contribute indirectly to Olsen-P through their mineralization to NaHCO_3_-P_i_ and NaOH-P_i_, respectively. The research results indicated that amino acid-enriched phosphate fertilizers can not only increase the yield but also do not increase the risk of P leaching, and have good environmental benefits compared with ordinary phosphate fertilizers. The addition of amino acids will promote the transformation of soil organic P to inorganic P to maintain the soil Olsen-P level. This study deepens the understanding of the effects of amino acid-enriched phosphate fertilizers on soil P pool characteristics, thus providing a basis for the development of new phosphate fertilizers.

## 1 Introduction

Phosphorus is an indispensable primary nutrient element in agricultural production, and the sufficient supply of phosphate fertilizers is crucial for ensuring high crop yields and excellent quality (Liu et al., 2015; Reinhard et al., 2017). However, due to the characteristics of water-soluble phosphate ions in phosphate fertilizers, such as their tendency to react with metal ions in the soil to form precipitates, be adsorbed by soil colloids, and be coated by iron and aluminum oxide films, they lose their activity. This leads to a large accumulation of phosphorus in the soil, with the utilization rate of phosphate fertilizers by crops in the current season being only 10%-25% (Zhang et al., 2008; Liu et al., 2016; Tonini et al., 2019). This low efficiency not only increases production costs but also causes water pollution, posing a threat to agricultural productivity and environmental sustainability. With the increasing depletion of phosphate rock resources, more scientific and sustainable practices must be adopted to reduce soil phosphorus fixation and improve the utilization efficiency of phosphate fertilizers to support crop growth and ensure food security (Mogollon et al., 2021). Previous studies have mainly included optimizing fertilization techniques, strengthening resource recycling strategies, and changing tillage technology schemes, etc (Zhang et al., 2023; Luo et al., 2024; McDowell et al., 2024; Zhong and Liang, 2024; McDowell and Haygarth, 2025). Although these techniques can reduce phosphorus (P) fixation and enhance uptake, they might also limit the interaction between plant roots and the soil, which could restrict overall P absorption (Meyer et al., 2022). In contrast to more unstable farming practices, fertilizer enhancers lower the necessary P input and align more effectively with the uptake patterns of crops, resulting in a significant improvement in phosphorus use efficiency (PUE) (Li et al., 2020; Guelfi et al., 2022). For example, Applying value-added phosphate fertilizers supplemented with glutamic acid in calcareous soil (pH = 8.07) can reduce the phosphorus fixation rate by 11.99%. Under low-phosphorus conditions, the wheat yield and the apparent utilization rate of phosphate fertilizers increase by 9.74% and 8.71% respectively (Li et al., 2013). Applying phosphate fertilizers supplemented with soybean fermentation broth in acid red soil (pH = 5.70) can promote maize photosynthesis, delay leaf senescence, and improve soil microbial diversity under low-phosphorus levels (Zhang et al., 2025).

The effectiveness of phosphate fertilizers is influenced not only by their formulation and compatibility with crops, but also by their interactions with various forms of soil phosphorus (Wei et al., 2024; Luo et al., 2026). The phosphorus components in soil are complex and diverse, ranging from ionic forms (e.g., HPO_4_^2-^ and H_2_PO_4_^-^) in solution to highly stable compounds (Rivard et al., 2016; Rodrigues et al., 2016; Liao et al., 2020). Therefore, more systematic indicators are needed to comprehensively evaluate the composition of soil phosphorus. Given the complexity and dynamics of soil phosphorus, qualitative and quantitative assessments often rely on methods for analyzing soil phosphorus fractions (Kizewski et al., 2011). Variations in adsorption and desorption capacities lead to different fates and migration mechanisms of different phosphorus fractions in soil (Yan et al., 2017; Mahmood et al., 2021; Azene et al., 2022). However, all fractions are in a complex equilibrium state, which collectively influences the availability of phosphorus (Bom et al., 2019; Soltangheisi et al., 2019). Therefore, elucidating the relative importance of different phosphorus fractions to soil available phosphorus (Olsen-P) is crucial for enhancing our understanding of soil phosphorus supply capacity and the risk of phosphorus leaching (Chen et al., 2022b).

Diammonium phosphate (DAP) is widely used due to its high solubility and high phosphorus content (IFASTAT, 2024), but in acidic paddy soils, it forms insoluble iron- and aluminum-phosphates (e.g., FePO_4_, AlPO_4_), thereby limiting phosphorus availability and crop uptake. Enhanced phosphorus fertilizers can inhibit phosphorus fixation, activate previously fixed phosphorus, and slow the transformation of available phosphorus into forms that are difficult to utilize (Leite et al., 2023). For example, adding amino acids to diammonium phosphate can increase available phosphorus content and lower soil pH (Sckefe et al., 2008; Boschetti et al., 2009; Sagar et al., 2023), promoting the conversion of Ca-P into Al-P and Fe-P forms that are more readily absorbed by plants. These formulations can also enhance soil enzyme activity (Senwo et al., 2007; Tian et al., 2016; Liu et al., 2019), facilitating the mineralization of organic phosphorus compounds. We assume that the amino acids of AP compete for metal-binding sites and dissolve fixed phosphorus to alleviate phosphorus fixation in acidic paddy soils.

This study utilized a soil column cultivation experiment to investigate the effects of value-added phosphate fertilizers with different amino acid addition levels (5% and 0.5%) on soil phosphorus pools, wheat yield, and phosphorus fertilizer use efficiency, The crop yield and soil Olsen-P and modified Hedley P fractions were investigated. The specific objectives of this study were (1) to investigate the effects of amino acid enhanced P fertilizers on the soil P fractions and availability variations in soil; (2) evaluate the effects of amino acid enhanced P fertilizers on rice yield and its components; (3) assess their impact on phosphate fertilizer utilization efficiency; and (4) to determine the contributions of different P fractions to soil Olsen-P. To provide data and theoretical basis for the research and development of plant-derived biostimulant fertilizer efficiency-enhancing carriers and their application in the development of new value-added phosphate fertilizer products.

## 2 Materials and methods

### 2.1 Tested soil and wheat varieties

The test soil was taken from the 0–20 cm topsoil of the three-year greenhouse in He county vegetable science and technology demonstration park, Anhui Province, China. It is classified as Typic paddy soil developed from Xiashu series parent material, with a medium - loam texture (Chinese Soil Taxonomy, CRGCST, 2001). Soils at depths of 0–20 cmwere collected, air - dried, mixed evenly, and sieved through a 2 mm sieve for later use. Physical and chemical properties of the soil are shown in **Table 1**:

**Table 1:** Basic physicochemical properties on soil of different layers.

Soil depth (cm)	pH	Organic matter (g/kg)	Total N (g/kg)	Available P (mg/kg)	Available K (mg/kg)
0~20	6.67	18.54	1.20	76.10	441.28

The wheat variety tested was ‘huaqimai 5’.

### 2.2 Test fertilizer

1. Amino acid synergist: Provided by the Institute of Agricultural Resources and Regional Planning, Chinese Academy of Agricultural Sciences. The total amino acid content is 20%, and the main components are glutamic acid, lysine, valine, and alanine.
2. Amino acid-enriched phosphate fertilizer (AP): The above amino acid synergist was added to diammonium phosphate at ratios of 5‰and 5% respectively, then mixed well, crushed, and sieved through a 1 mm sieve. Finally, AP0.5 (diammonium phosphate enriched with 0.5% amino acid) and AP5 (diammonium phosphate enriched with 5% amino acid) were obtained. Solid-state ^13^C nuclear magnetic resonance was used to collect the spectra of the enriched phosphate fertilizers with different amino acid addition amounts, and the spectra were integrated to obtain the relative contents of various carbon-containing functional groups in the samples (**Table 2**).
3. Common phosphate fertilizer (P): Use the same diammonium phosphate as in (2) and repeat the operations in (2), but without adding the amino acid synergist. After mixing, grinding, and sieving, the obtained diammonium phosphate (P) is used as the control for (2).

**Table 2:** Relative proportions of carbon types determined by CP/TOSS and diploar-dephased techniques in different enhanced phosphate fertilizer with different amounts of amino acid addition.

Tested amino acid enhanced P fertilizers	Alkyl C	O-alkyl C	Aromatic C	Aromatic C–O	COO/N–C=O	Ketone/ aldehyde
AP0.5	4.74	5.37	51.37	14.15	22.54	1.87
AP5	5.61	5.67	51.75	14.23	20.81	1.74

The basic physical and chemical properties of all tested fertilizers are shown in **Table 3**.

**Table 3:** Additive proportion of amino acid and P_2_O_5_contents in prepared phosphorous fertilizer.

Treatment	Amino acid (%)	P^2^O^5^ content (%)	pH
P	0	52.79	4.13
AP0.5	0.5	52.51	4.13
AP 5	5	52.12	4.13

### 2.3 Experimental design

Five treatments were set up in the experiment: (1) no - fertilization control (CK), (2) control without phosphorus fertilizer (NK), (3) application of diammonium phosphate (P), (4) application of 0.5% amino - acid - enhanced diammonium phosphate (AP0.5), and (5) application of 5% amino - acid - enhanced diammonium phosphate (AP5). Each treatment was replicated 6 times.

The experiment adopted the soil column cultivation method. PVC pipes with an inner diameter of 25 cm and a height of 100 cm were selected and fully buried in the soil. The upper opening was 3 cm above the ground to prevent surface runoff from precipitation from flowing in. The lower end was not sealed and was in direct contact with the natural soil to simulate the field cultivation state (Because the columns were open at the base and contacted natural soil, capillary rise, drainage, and solute exchange were not fully controlled. This design limits mass-balance and leaching interpretations and may reduce comparability with closed-column or field studies). Each soil column was filled with 50 kg of air - dried soil, and the fertilization amount was calculated based on the weight of the 0–30 cm soil layer. Among them, different phosphorus application levels were designed for amino - acid - enhanced phosphate fertilizer and ordinary monoammonium phosphate according to the principle of equal total phosphorus input, with a P_2_O_5_ application rate of 0.15 g/kg soil. Nitrogen and potassium fertilizers were designed according to the principle of sufficient supply. Urea (containing 46% N) was selected as the nitrogen fertilizer, with an application rate of 0.2 g N/kg soil; potassium sulfate (K_2_O 63.0%) was selected as the potassium fertilizer, with an application rate of 0.3 g K_2_O/kg soil. Both nitrogen and potassium fertilizers were mixed and applied as base fertilizers into the 0–30 cm soil layer at one time. The experiment was sown on November 9, 2024. Well - selected, uniform and plump wheat seeds were used, with 36 seeds sown in each column. Thinning was carried out during the seedling stage, leaving 12 plants in each soil column. At the beginning of the experiment, the soil moisture content was 18.6%, and it was maintained at approximately 75% of the field capacity through weighing method. Each experimental column received 0.5 liters of water during each irrigation throughout the wheat growth period; in addition, an additional 3 liters of water was applied on wheat green - turning stage (April 12, 2025) and the flag - leaf stage (May 1, 2025). An open bottom design was adopted to facilitate drainage, and a double-layer permeable gauze + 3cm thick coarse quartz sand filter layer was implemented at the bottom of the experimental column. The accumulated water at the bottom was regularly cleaned to maintain ventilation and avoid soil waterlogging and root hypoxia. The wheat was harvested for yield measurement on June 18, 2025, and was managed according to conventional cultivation measures during the growth period.

### 2.4 Soil and plant sampling and analysis

Plant samples were collected at the maturity stage of wheat and were systematically categorized into three distinct parts: stem, leaf, and grain. To ensure proper handling and identification, these samples were placed in adequately labeled mesh bags and subsequently transported to the laboratory for further analysis. Within the grain samples, air-drying was performed, and the number of spikes was meticulously recorded. Following this, the grains were threshed and weighed to obtain precise measurements. Prior to any additional procedures, the samples underwent a rinsing process with both tap water and ultra-pure water, after which fresh weights were documented. For the purpose of conducting subsequent analyses, samples were oven-dried initially at a temperature of 105 °C for 30 minutes, followed by a temperature reduction to 75 °C until a constant weight was achieved. Once fully dried, the samples were weighed again before being ground for further analytical assessments. The concentration of total phosphorus (P) was determined using an established protocol involving H_2_SO_4_ and H_2_O_2_ digestion (conducted with a DigiBlock ST36, LabTech, USA), and the resulting samples were analyzed through a continuous flow analyzer (San++, Skalar, Netherlands). Soil samples were collected at various stages of wheat development, specifically during tillering, jointing, anthesis, and maturity. The topsoil samples, taken from a depth of 0–20 cm, were air-dried to facilitate handling, and then they were sieved through nylon screens with mesh sizes of 2 mm and 0.15 mm, following the removal of any plant roots and gravel. This procedure was essential for accurately analyzing the properties of the soil. Available phosphorus was extracted from the soil using a 0.5 mol L^-1^ solution of NaHCO_3_ and was subsequently measured through UV-visible spectrophotometry (Cary 3500, Agilent, USA), utilizing a molybdenum-antimony resistance colorimetric method. The analysis of phosphorus fractions was carried out in accordance with the modified Hedley fractionation method proposed by Wei (Wei et al., 2024), which allows for a detailed classification of phosphorus based on its bioavailability. The phosphorus fractions were categorized into three main groups: labile phosphorus, which includes H_2_O-P, NaHCO_3_-P_i_, and NaHCO_3_-P_o_; moderately labile phosphorus, consisting of NaOH-P_i_ and NaOH-P_o_; and stable phosphorus, comprising HCl-P and residual phosphorus.

### 2.5 Data processing and analysis

Plant phosphorus accumulation (g/pot) = Plant phosphorus content × Plant biomass;

Accumulative apparent efficiency of phosphorus (%) = (Total phosphorus uptake by plants with phosphate fertilizer application - Total phosphorus uptake by plants without phosphate fertilizer application)/Amount of phosphate fertilizer applied × 100%;

physiological use efficiency of phosphorus (%) = (Crop yield in phosphorus - applied treatment - Crop yield in non - phosphorus - applied treatment)/(Phosphorus uptake by plants in phosphorus - applied treatment - Phosphorus uptake by plants in non - phosphorus - applied treatment);

Agronomic efficiency of phosphorus (kg/kg) = Grain yield under phosphate fertilizer application/Phosphate application rate;

Partial factor productivity of phosphorus (kg/kg) = (Grain yield with phosphate fertilizer application - Grain yield without phosphate fertilizer application)/Phosphate application rate;

In this study, the experimental data were evaluated utilizing one-way ANOVA alongside the least significant difference test, while Duncan’s new multiple range test was implemented for conducting multiple comparisons (with statistical significance defined at *P* < 0.05). To ascertain the connections between Olsen-P and the various phosphorus fractions, Pearson correlation analysis was conducted. The significance of phosphorus scores in explaining Olsen-P was outlined using partial least squares regression (PLSR). Furthermore, path analysis was utilized to investigate the causal links among different soil phosphorus pools, as well as to differentiate between the direct and indirect impacts of these relationships. All statistical evaluations were carried out using R version 4.2.1 (RStudio, Boston, Massachusetts, USA). Figures were produced with Origin 2022 (OriginLab Corporation, Northampton, Massachusetts, USA). For each response variable, ANOVA residuals were evaluated using the Shapiro-Wilk test and Q-Q plots, and homogeneity of variance was assessed using Levene’ s test. Following a significant omnibus ANOVA, treatment means were compared using Tukey’ s HSD with family-wise α = 0.05.

## 3 Results

### 3.1 Effect of amino acid enhanced phosphorus fertilizer on soil available phosphorus

**Figure 1:**
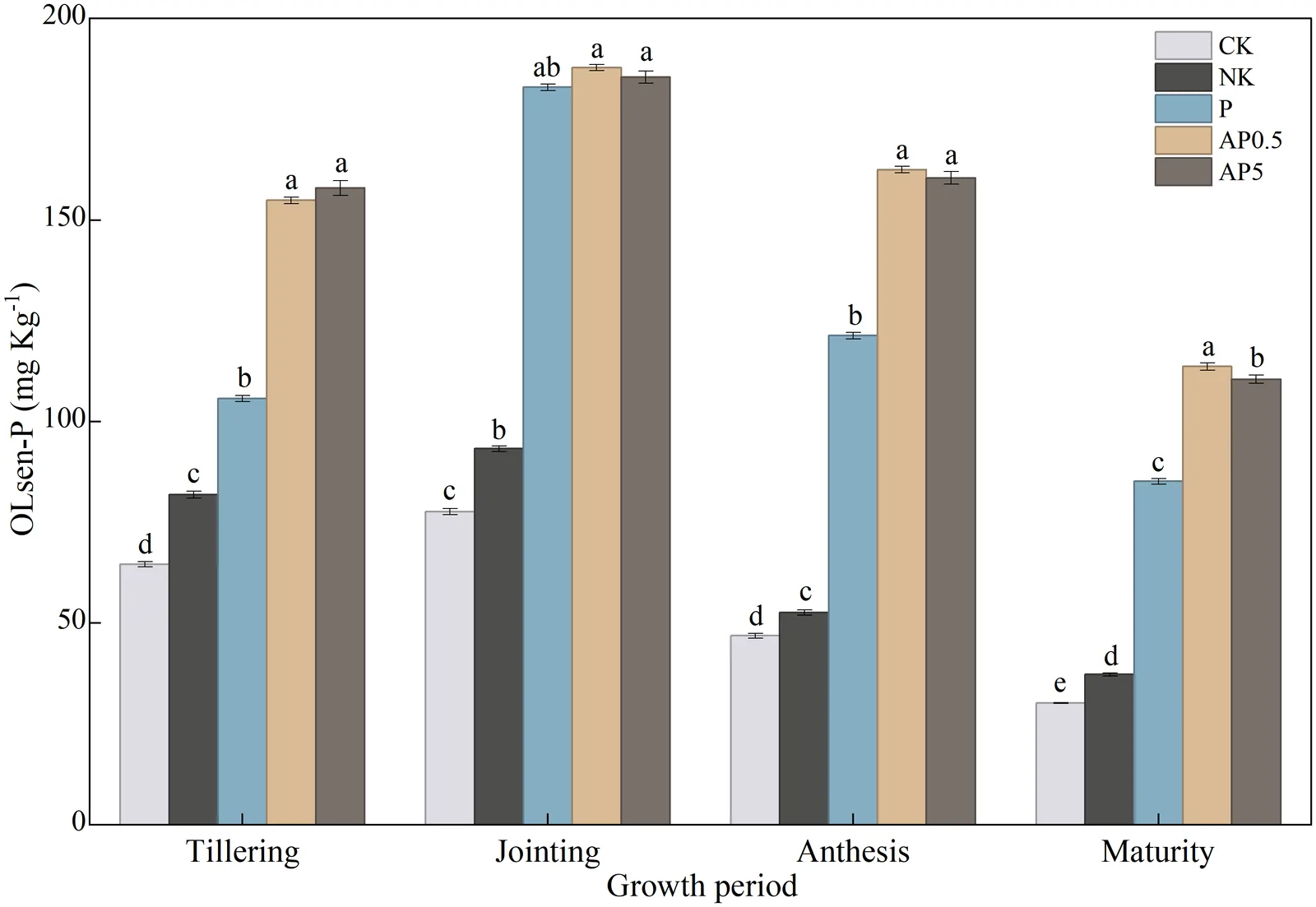
Effect of amino acid enhanced phosphate fertilizer on soil available phosphorus. Different lowercase letters indicate significant differences among treatments at *P* < 0.05.

Soil available phosphorus is an important indicator for evaluating soil phosphorus availability. As shown in **Figure 1**, the application of phosphate fertilizer significantly increased the soil available phosphorus content (*P* < 0.05), showing an overall trend of increasing first and then decreasing from the seedling stage to the maturity stage, which is consistent with the nutrient release law of phosphorus in the soil. Overall, different phosphate fertilizer treatments have different effects on soil phosphorus availability. Relative to P, AP0.5 and AP5 increased Olsen-P by 46.54-49.45% at tillering, 1.39-2.66% at jointing, 32.24-33.93% at anthesis, and 29.76-33.45% at maturity. The small jointing-stage percentage reflects the high Olsen-P value already observed under P at that stage. Except for the tillering stage, the difference was most significant under the AP0.5 treatment in other stages. Only at the maturity stage, the soil available phosphorus content under the AP0.5 treatment was significantly higher than that under the AP5 treatment, and there were no significant differences between different amino acid addition amounts in other periods.

### 3.2 Effect of amino acid enhanced phosphorus fertilizer on soil phosphorus composition

**Figure 2:**
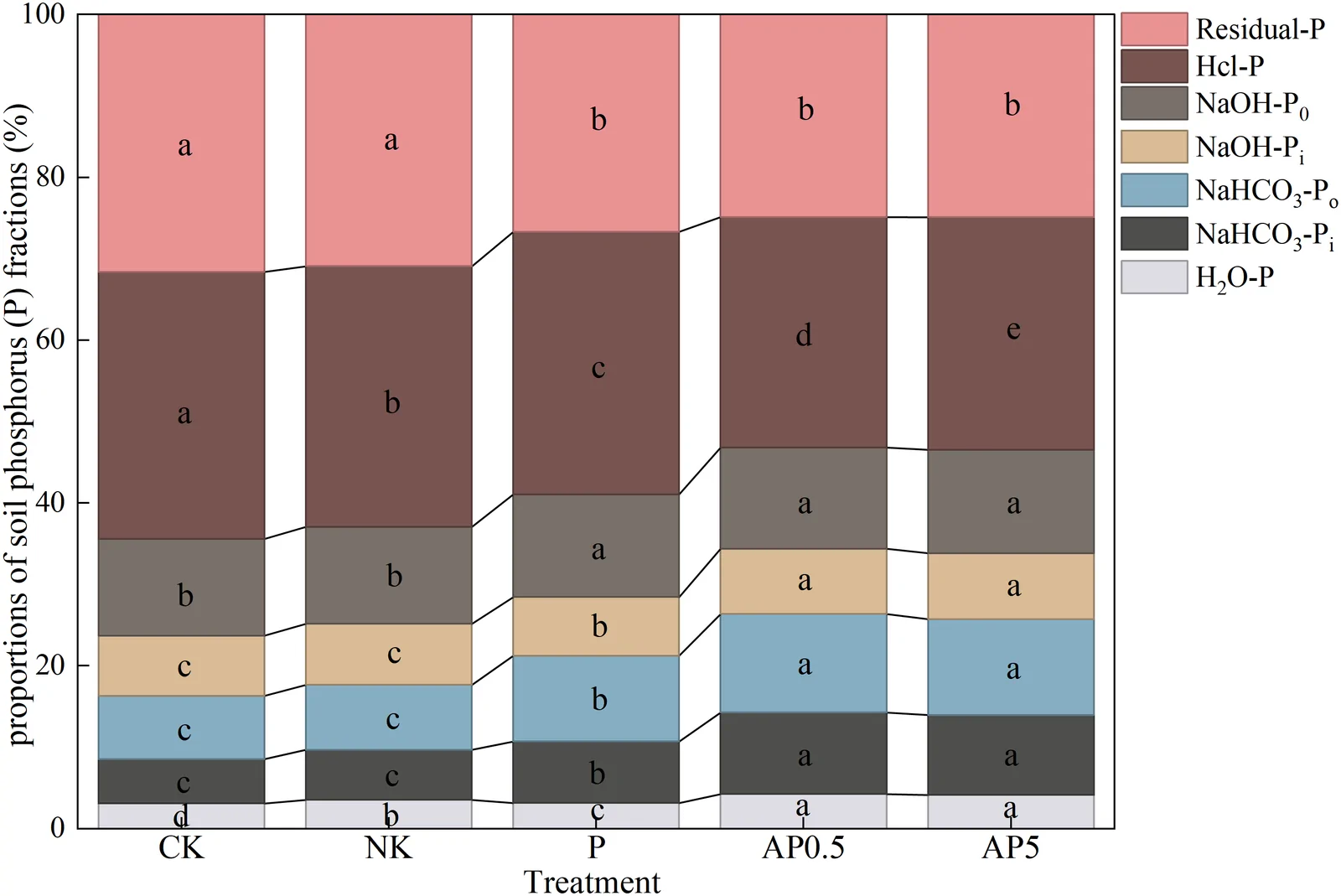
Effect of amino acid enhanced phosphate fertilizer on soil phosphorus composition. Area-normalized yield equivalents were calculated from the column surface area (0.0491 m^2^) and are provided only for agronomic context; they should not be interpreted as field-scale yield estimates. Different lowercase letters indicate significant differences among treatments at *P* < 0.05.

Crops vary in their ability to absorb different phosphorus (P) fractions in the soil. Among them, labile P is more easily absorbed by crops due to its high mineralization potential, while stable P is readily fixed in the soil, making it difficult for crop roots to take up. The percentages of different P fractions in the total P content of wheat rhizosphere soil are presented in **Figure 2**. In all treatments, stable P fractions (HCl - P, Residual - P) dominated the soil P fractions, followed by moderately labile P (NaOH - P_i_ and NaOH - P_o_), while the relative proportion of labile P (H_2_O - P, NaHCO_3_- P_i_ and NaHCO_3_- P_o_) was relatively low. The proportions of different P fractions varied among treatments. H_2_O - P and NaHCO_3_- P in the soil were preferentially absorbed by plants under P - deficient conditions. In contrast, HCl - P and Residual - P are relatively stable P forms. Compared with the treatment of common phosphate fertilizer (P), the contents of H_2_O - P, NaHCO_3_- Pi and NaHCO_3_ - Po in the treatments of amino - acid - enhanced phosphate fertilizers (AP0.5 and AP5) increased by 0.99% - 1.11%, 2.27% - 2.46% and 1.23% - 1.54% respectively, with significant differences. There were no significant differences between treatments with different amino - acid addition rates, indicating that amino acids promoted the transformation of moderately labile P to labile P in the soil, thus activating the soil P pool. Compared with P, the contents of HCl - P and Residual - P in the AP0.5 and AP5 treatments decreased by 3.71% - 3.94% and 1.76% - 1.81% respectively, with the most significant difference observed at the 0.5% addition rate, suggesting that the change in soil residual P is closely related to the soil P content.

### 3.3 Effect of amino acid enhanced phosphorus fertilizer on phosphorus absorption and utilization of Wheat

**Figure 3:**
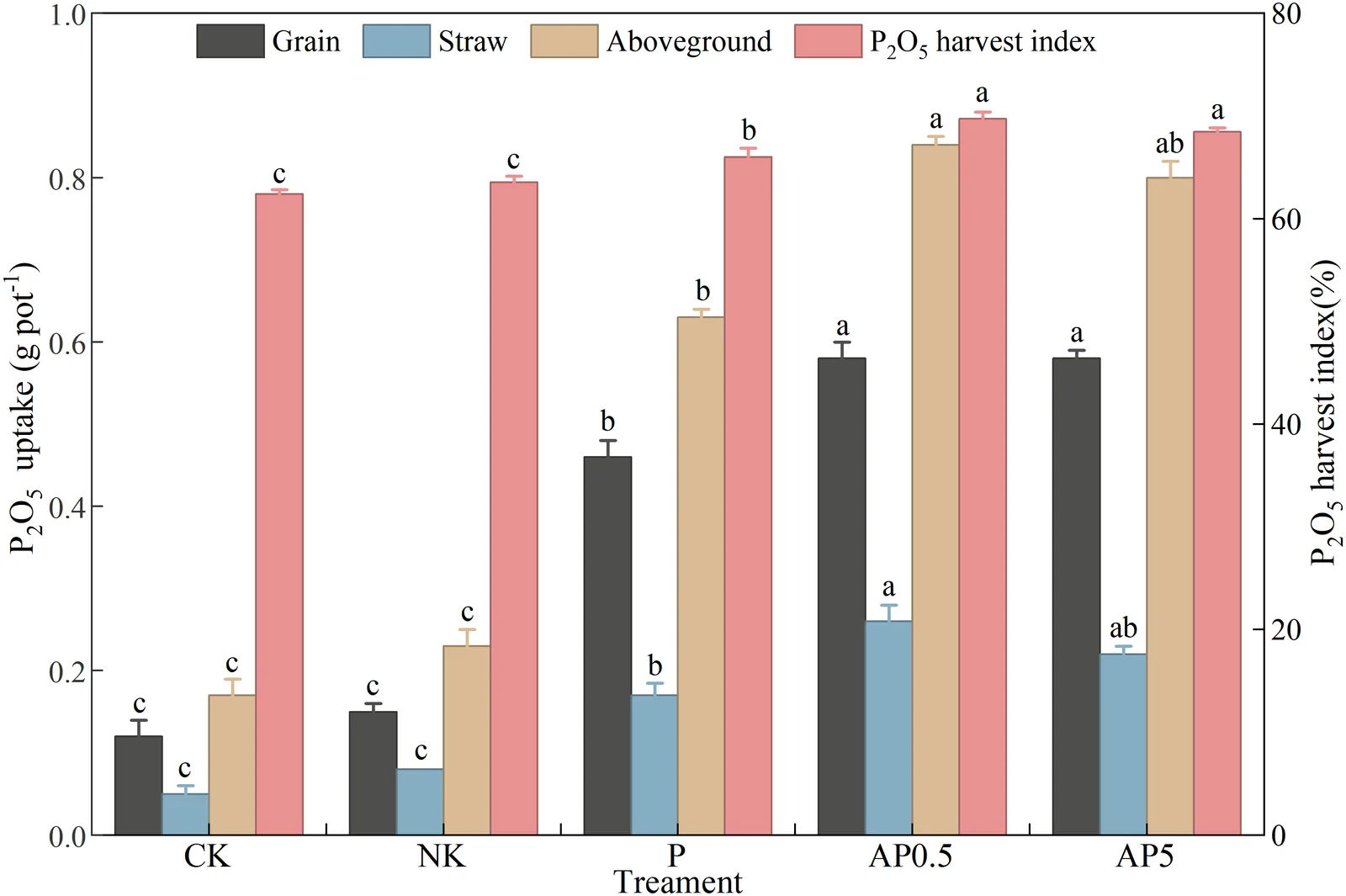
Effect of amino acid enhanced phosphate fertilizer on phosphorus absorption and utilization of wheat. Different lowercase letters indicate significant differences among treatments at *P* < 0.05.

It can be seen from **Figure 3** that the application of phosphate fertilizer can significantly promote the absorption of phosphorus by wheat grains, straws, and above - ground parts. The increase in phosphorus uptake by wheat grains and straws is more obvious under the treatment of amino - acid enhanced phosphate fertilizer compared with that of ordinary phosphate fertilizer. Compared with the ordinary phosphate fertilizer (P), the phosphorus uptake by wheat grains, straws, and above - ground parts under the treatments of amino - acid enhanced phosphate fertilizers (AP0.5 and AP5) increased by 26.09%, 29.41% - 52.94%, and 26.98% - 33.33%, respectively, and there were significant differences except for the AP5 treatment. The phosphorus harvest index increased significantly by 2.45% - 3.72%. It can also be seen from **Figure 3** that different addition amounts of amino acids have no effect on the phosphorus uptake by grains, and the phosphorus uptake by wheat straws shows a decreasing trend with the increase in the addition ratio of amino acids in the enhanced phosphate fertilizer.

Further analysis of the phosphorus content in wheat plants (**Table 4**) reveals that the average phosphorus content in wheat grains under the treatment of amino acid - enhanced phosphate fertilizer is slightly lower than that under the treatment of ordinary phosphate fertilizer. Compared with the P treatment, the phosphorus content in wheat grains under the AP0.5 and AP5 treatments decreased by 0.06% and 0.01% respectively. This may be due to the dilution effect caused by the increase in crop yield after applying the enhanced - efficiency phosphate fertilizer compared to monoammonium phosphate. However, the average phosphorus content in wheat straw under the amino acid - enhanced phosphate fertilizer treatments is higher than that under the ordinary phosphate fertilizer treatment. The average phosphorus content in straw under the amino acid - enhanced phosphate fertilizer treatments (AP0.5 and AP5) increased by 0.06% and 0.05% respectively compared to the ordinary phosphate fertilizer treatment (P).

**Table 4:** P contents of grain and straw of wheat (%).

Treatment	P contents (%)	
	Grain	Straw
CK	0.33 ± 0.02c	0.11 ± 0.01d
NK	0.36 ± 0.01b	0.15 ± 0.03c
P	0.38 ± 0.01a	0.18 ± 0.01b
AP0.5	0.32 ± 0.02cd	0.24 ± 0.02a
AP5	0.37 ± 0.01ab	0.23 ± 0.01ab

Values followed by different lowercase letters in a column mean significant difference among treatments at 5% level.

### 3.4 Effect of amino acid enhanced phosphorus fertilizer on wheat phosphate fertilizer utilization

**Figure 4:**
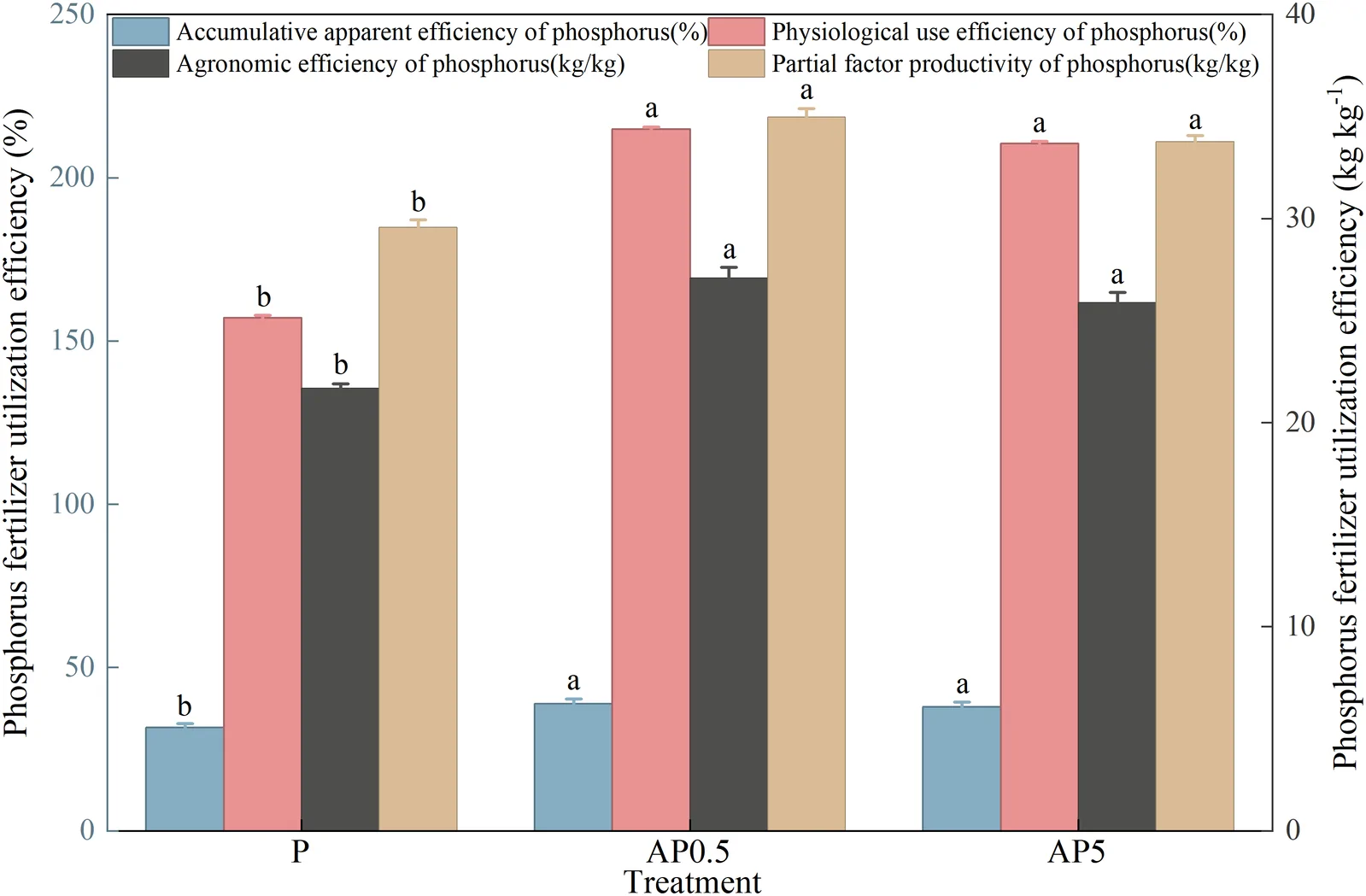
Phosphate fertilizer efficiency of wheat under different treatments. Different lowercase letters indicate significant differences among treatments at *P* < 0.05.

By analyzing the utilization efficiency of phosphate fertilizer in wheat (**Figure 4**), it can be seen that compared with the common phosphate fertilizer treatment (P), the amino acid - enhanced phosphate fertilizers (AP0.5 and AP5) can improve the Accumulative apparent efficiency of phosphorus, physiological use efficiency of phosphorus, Agronomic efficiency of phosphorus, and Partial factor productivity of phosphorus. Compared with the common phosphate fertilizer treatment (P), the Accumulative apparent efficiency of phosphorus and physiological use efficiency of phosphorus fertilizer in the amino acid - enhanced phosphate fertilizer treatments (AP0.5 and AP5) increased by 6.34 - 7.24 and 53.29 - 57.75 percentage points respectively, and the partial productivity and agronomic efficiency of phosphate fertilizer increased by 14.20% - 18.26% and 19.36% - 24.90% respectively, with all differences being significant. However, the differences between the treatments with different amino acid addition amounts (AP0.5 and AP5) were not significant.

### 3.5 Effects of amino acid enhanced phosphorus fertilizer on wheat yield and its components

The effects of different treatments on wheat yield and its components are shown in **Table 5**. As can be seen from the table, compared with the treatment without phosphate fertilizer (NK), the application of phosphate fertilizers (P, AP0.5, AP5) increased the wheat grain yield and biomass yield by 275.30% - 343.77% and 191.10% - 218.75% respectively, with a significant yield - increasing effect (*P* < 0.05). Further analysis of the yield - forming factors shows that, compared with the treatment without phosphate fertilizer (NK), the application of phosphate fertilizers (P, AP0.5, AP5) increased the effective spike number, grain number per spike and 1000 - grain weight of wheat by 106.17% - 146.32%, 42.11% - 62.08% and 14.88% - 19.10% respectively, with significant differences (*P* < 0.05). Among the phosphate - applying treatments, the 1000 - grain weight of wheat under the treatment of amino - acid - enhanced phosphate fertilizers (AP0.5, AP5) was 1.76% - 3.67% higher than that under the treatment of common phosphate fertilizer (P), with a significant difference. Compared with P, the treatments of AP0.5 and AP5 could increase the effective spike number and grain number per spike of wheat to a certain extent, but there was no significant difference among the phosphate - applying treatments. Moreover, there was no significant difference in the responses of wheat yield and its components to the added amino - acid content in the enhanced phosphate fertilizers.

**Table 5:** Wheat grain yield and its components under different treatments.

Treatment	Grain yield (g/pot)	Biological yield (g/pot)	Effective spike (No./pot)	Number of grains (No./spike)	1000-kernel weight 1001-(g)
CK	15.82 ± 0.35c	41.53 ± 1.36c	16.00 ± 0.36b	22.76 ± 0.58b	34.77 ± 1.24c
NK	17.73 ± 0.42c	46.39 ± 1.13c	20.25 ± 0.26b	24.79 ± 0.39b	39.10 ± 1.33c
P	66.54 ± 0.89b	135.04 ± 0.81b	41.75 ± 0.58a	35.23 ± 0.51a	44.92 ± 1.36b
AP0.5	78.68 ± 1.38a	147.87 ± 1.60a	49.88 ± 0.45a	40.18 ± 0.32a	46.57 ± 1.14a
AP5	75.99 ± 1.72a	140.07 ± 1.47a	46.52 ± 0.88a	38.36 ± 0.49a	45.71 ± 1.50ab

Area-normalized yield equivalents were calculated from the column surface area (0.0491 m²) and are provided only for agronomic context; they should not be interpreted as field-scale yield estimates. Values followed by different lowercase letters in a column mean significant difference among treatments at 5% level.

### 3.6 Relationship between different amino acid enhanced phosphorus fertilizer and wheat yield and phosphate fertilizer utilization efficiency

**Figure 5:**
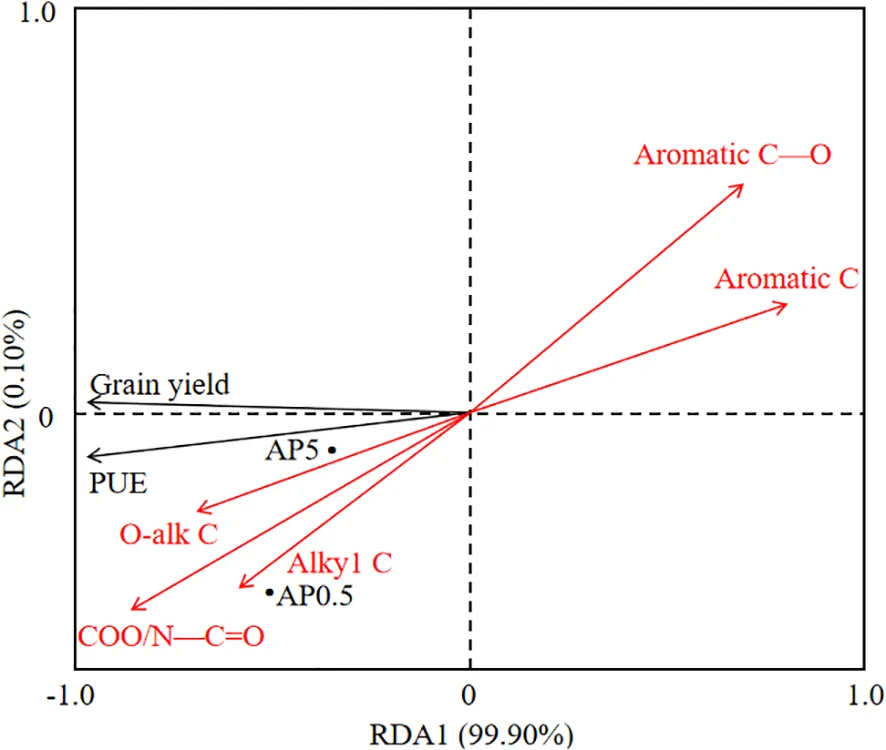
Redundancy analysis between functional groups of different enhanced phosphate fertilizer with different amounts of amino acid addition and wheat grain yield and phosphate fertilizer recovery efficiencies.

Combined with the attribution of each carbon-containing functional group in the solid-phase ^13^C nuclear magnetic resonance characterization results of value-added phosphate fertilizers with different amino acid addition amounts in **Table 2**, the main functional groups (alkyl carbon, oxygen-alkyl carbon, aromatic carbon, aromatic C—O, carboxyl/amide carbon) were selected as explanatory variables, and the wheat grain yield and phosphate fertilizer utilization rate were used as response variables for redundancy analysis to explore the relationship between the functional groups of value-added phosphate fertilizers with different amino acid addition amounts and wheat yield and phosphate fertilizer utilization rate. As shown in **Figure 5**, the first two principal axes (RDA1 and RDA2) explained 99.90% and 0.10% of the total variables in the model, respectively. The wheat grain yield and phosphate fertilizer utilization rate were positively correlated with alkyl carbon, oxygen-alkyl carbon, and carboxyl/amide carbon, and the order of the correlation magnitude was oxygen-alkyl carbon > carboxyl/amide carbon > alkyl carbon, and negatively correlated with aromatic carbon and aromatic C—O. AP0.5 has more carboxyl/amide carbon structures. The ^13^C NMR profiles of AP0.5 and AP5 were broadly similar; AP0.5 contained a slightly higher proportion of carboxyl/amide C (22.54% vs. 20.81%). Because only two formulations were characterized, these differences are descriptive and cannot be related statistically to yield or P-use efficiency.

### 3.7 Relationships between Olsen-P and P fractions

**Figure 6:**
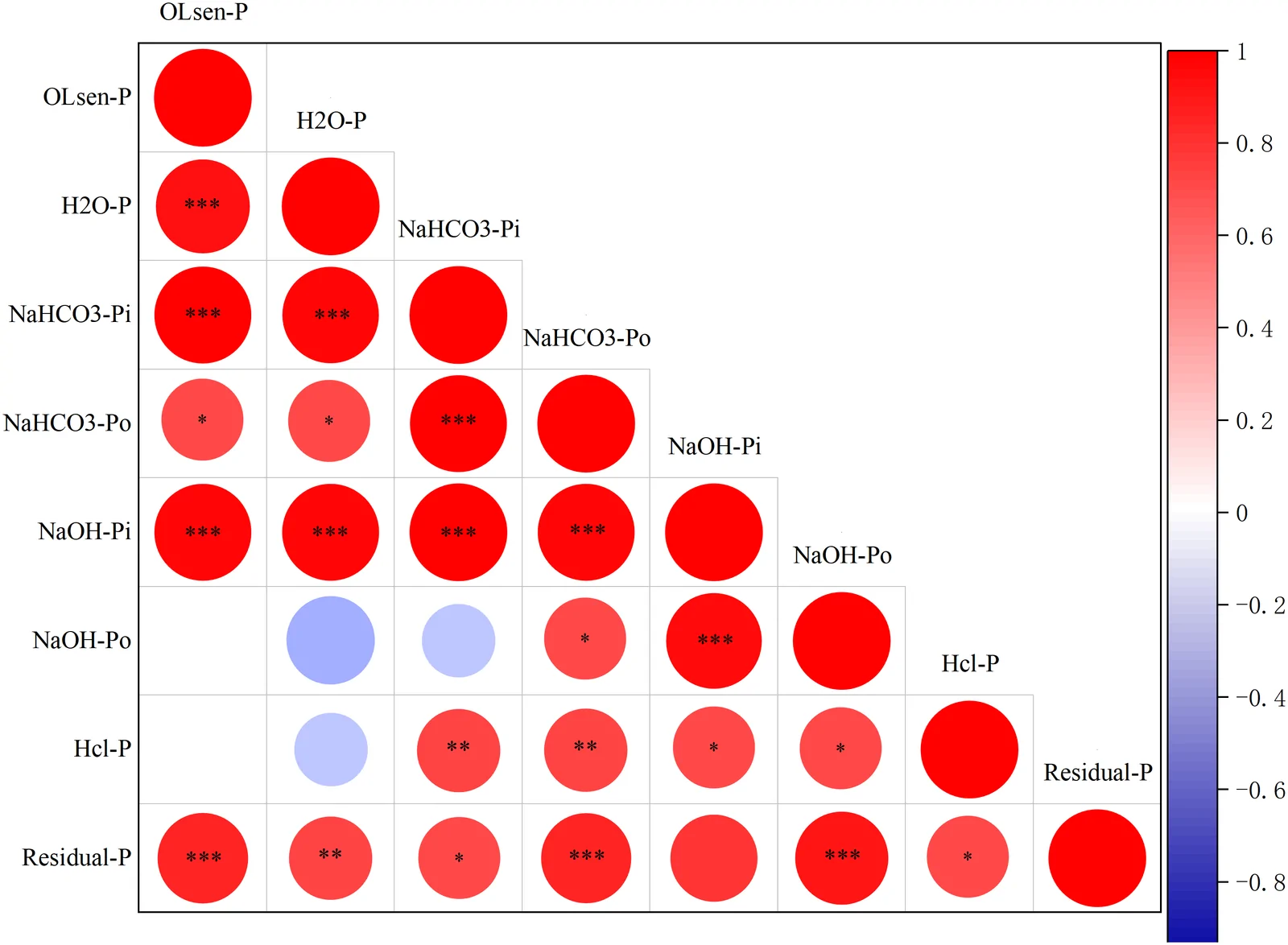
The relationship coefficients between Olsen-P and the various phosphorus fractions. Significance levels are denoted as *P < 0.05, **P < 0.01, and ***P < 0.001.

**Figure 7:**
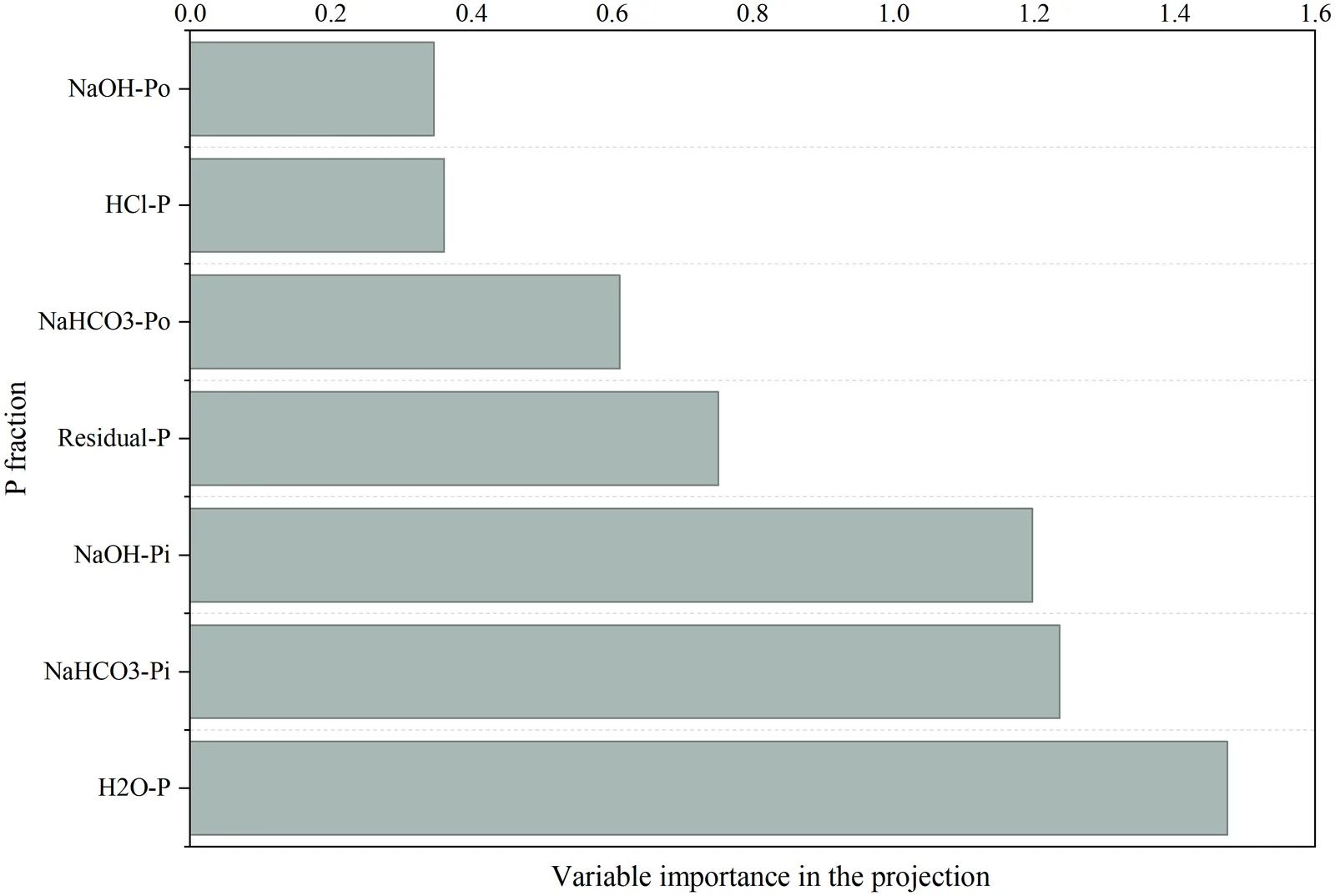
Illustrates the projection of variable importance between the Olsen-P and P fractions. The height of the bars represents the value associated with the projection of variable importance.

The correlation analysis between soil available phosphorus (Olsen-P) and different phosphorus forms is shown in **Figure 6** (*R* > 0.6). Olsen-P showed a significant positive correlation with most soil phosphorus components, and the correlation coefficients between Olsen-P and H_2_O-P, NaHCO_3_-Pi, and NaOH-P_i_ were relatively high (*R* > 0.6). Partial least squares regression (PLSR) analysis (**Figure 7**) showed thatH_2_O-P、NaHCO_3_-P_i_ and NaOH-P_i_ had the highest variable importance in the projection, with values greater than 1, suggesting that these P fractions played the most important role in explaining Olsen-P (**Figure 7**).

**Figure 8:**
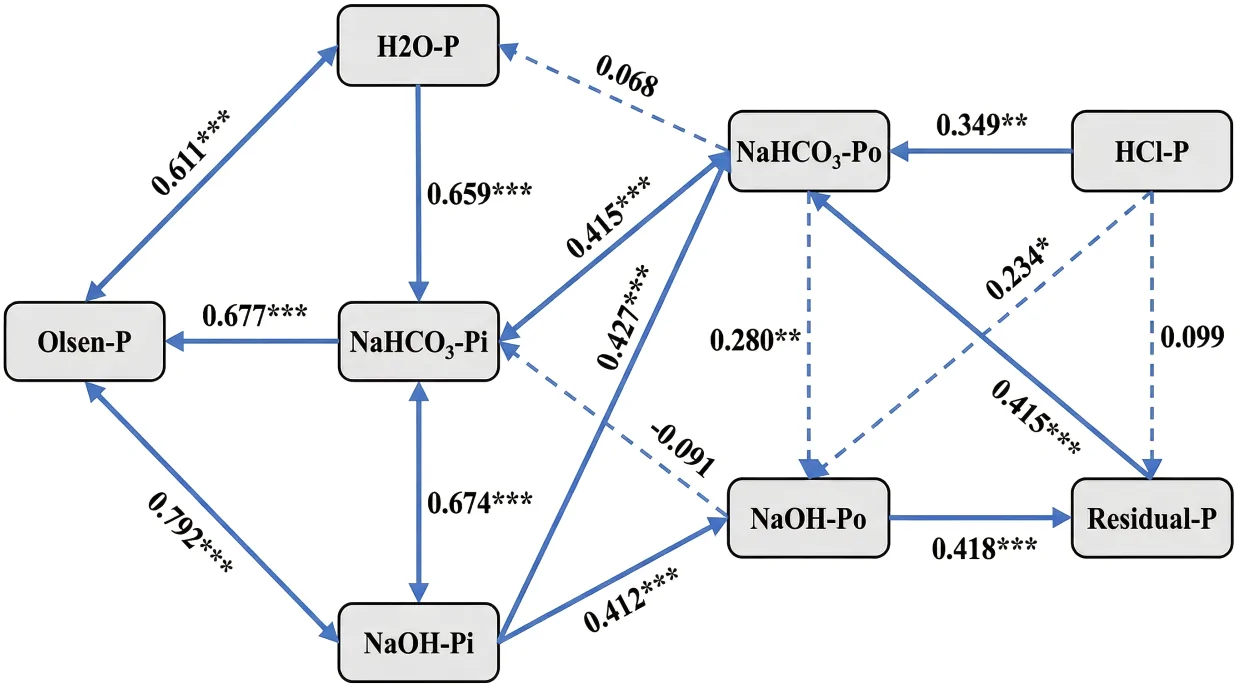
Analysis of path relationships involving Olsen-P and various phosphorus fractions. Solid arrows indicate significant effects and directions (*P* < 0.05), while dashed arrows illustrate insignificant effects and directions (*P* ≥ 0.05). The numbers linked to the lines reflect the standardized coefficients for the paths. Levels of significance are represented as *P < 0.05, **P < 0.01, and ***P < 0.001.

Path analysis revealed that H_2_O-P, NaHCO_3_-P_i_, and NaOH-P_i_ had direct effects on Olsen-P, whereas NaHCO_3_-P_o_ and NaOH-P_o_ had mediating effects through NaHCO_3_-P_i_ and NaOH-P_i_ (**Figure 8**). Pearson correlations and PLSR identified H_2_O-P, NaHCO_3_-P_i_, and NaOH-P_i_ as fractions most strongly associated with Olsen-P; these analyses do not establish causal pathways among P pools.

## 4 Discussion

### 4.1 Effect of amino acid enhanced phosphorus fertilizers on Olsen-P and soil P turnover

Soil available phosphorus is an important basis for evaluating the soil phosphorus supply capacity and determining the phosphorus application rate, and it is commonly used to evaluate the availability of soil phosphorus. The results of this study indicate that the treatments with amino acid - enhanced phosphate fertilizers have differential effects on the soil available phosphorus content. Compared with ordinary phosphate fertilizers, the application of 0.5% amino acid - enhanced phosphate fertilizer has the most significant effect on increasing the soil available phosphorus content at different growth stages of wheat. The reasons may be as follows. On the one hand, amino acids, when applied to the soil as activators, can complex with Ca^2+^, Fe^3+^, and Al^3+^, reducing the combination of cations with P to form precipitates, thereby activating soil phosphorus and improving phosphorus availability (Yang et al., 2019; Zhang et al., 2024). This is consistent with the research results of Fu Wenjie et al (Fu et al., 2021). and Cheng Lin et al (Cheng et al., 2024). On the other hand, amino acids have colloidal properties and can compete with phosphates in fertilizers for adsorption sites on the surface of soil minerals and prevent the precipitation of phosphorus in the solution (Johnson et al., 2006). In addition, the proton hydrogen carried by amino acids can not only activate the fixed phosphorus in the soil but also form amino acid - metal ion complexes with metal ions, enhancing the solubility of phosphorus in fertilizers, reducing the fixation of phosphorus in the soil and fertilizers, and enhancing the mobility of phosphorus, thus improving phosphorus availability (Quaggiotti et al., 2004; Peng and Deng, 2017). However, the improvement degrees of soil phosphorus availability by different amino acid addition amounts vary, which may be related to the obvious threshold of the promoting effect of amino - acid - based biostimulants (Ocwa et al., 2024). The numerical advantage of AP0.5 over AP5 suggests diminishing returns rather than proven inhibition at 5%. Because only two rates were tested, the optimum dose cannot be identified; experiments spanning lower and intermediate rates are required.

The changes in the soil phosphorus (P) pool are influenced by the type and amount of phosphate fertilizers applied. This study shows that the P in the tested soil is mainly composed of moderately labile P, which may be attributed to the subtropical monsoon climate and acidic soil conditions. Numerous studies have indicated that water-soluble P is rapidly converted into insoluble P, and soil cations fix this P, forming moderately recalcitrant forms. In acidic soils, the applied P tends to be adsorbed and fixed by iron oxides, aluminum oxides, and clay minerals (Wang et al., 2015; Peng et al., 2025). The application of phosphate fertilizers significantly increased various soil P fractions, which is consistent with previous research results (Ando et al., 2022; Wei et al., 2024; Luo et al., 2026). Treatment-specific soil pH was not measured during crop growth; therefore, pH-mediated P mobilization remains a hypothesis derived from previous studies rather than a mechanism demonstrated here. Specifically, the application of phosphate fertilizers increased the concentration and proportion of labile P (including H_2_O - P, NaHCO_3_ - P_i_, and NaHCO_3_ -P_o_) and moderately labile P (such as NaOH-P_o_) (Farhan et al., 2021; Peng et al., 2025). Notably, the treatment with ordinary phosphate fertilizers did not significantly increase the H_2_O-P level but increased the NaHCO_3_- P_i_ and NaHCO_3_-P_o_ levels. Meanwhile, it had no significant effect on HCl - soluble P and Residual - P. In contrast, the amino - acid - enriched phosphate fertilizer maintained a high level of labile P in the soil for a long time by regulating the P release rate. As crop growth progressed, soil P shifted from a more labile pool to moderately labile forms. Increases in NaOH - P_i_, NaOH - P_o_, and HCl - P and decreased Residual - P resulted in enhanced P activation and improved soil P availability. The above results indicate that different phosphate fertilizers have significant differences in their effects on soil P fractions. Water - soluble fertilizers are rapidly converted into labile P (Labile P, H_2_O - P, NaHCO_3_- P_i_, and NaHCO_3_- P_o_) and may then be transformed into moderately stable P due to the fixation by soil particles (Wei et al., 2024). Both organic and inorganic P are involved in the plant P uptake process and can be transformed through biological pathways (Wang et al., 2025). Plants can only directly utilize low - stability P, while moderately stable P can serve as a potential reservoir for low - stability P. This study shows that the amino - acid - enriched phosphate fertilizer can promote the transformation and storage of P into highly and moderately stable P forms, thus meeting the long - term P requirements of crops. This strategy conforms to the principle of “achieving greater benefits with fewer resources” and is in line with the goals of sustainable agricultural development. The initial Olsen-P concentration was 76.10 mg kg^-1^, indicating a high-P soil. Thus, the results address P-pool redistribution and crop response under elevated background P and should not be generalized to P-deficient soils; the degree of P saturation was not measured.

### 4.2 Effect of new P fertilizers on wheat grain yield and utilization rate of phosphate fertilizer

As a key limiting factor affecting crop productivity, the rational application of phosphorus can significantly increase crop yields. Phosphate fertilizers can effectively improve the yield - forming factors of rice by increasing the number of panicles, the number of grains per panicle, and the seed - setting rate (Fageria et al., 2015; Wei et al., 2024). In this study, the application of phosphate fertilizers significantly increased the wheat yield by 275.30% - 343.77%. Notably, the increase in yield was mainly attributed to a 106.17% - 146.32% increase in the number of effective panicles, indicating an improvement in tillering ability. This is consistent with the study by Pu Zitian et al (Pu et al., 2022). In this study, the yields of AP0.5 and AP5 were significantly higher than that of P. This might be because the amino - acid - enhanced phosphate fertilizers are rich in functional groups such as carboxyl, phenolic hydroxyl, hydroxyl, methoxy, amino, keto, and quinone groupsas “binding sites” that can selectively form stable, soluble complexes (chelates) with free metal ions such as Ca^2+^, Mg^2+^, Fe^3+^, and Al^3+^in the soil (Yang et al., 2019; Zhang et al., 2024). This process prevents these ions from reacting with subsequently applied P fertilizers, thereby reducing the likelihood of precipitate formation and minimizing P fixation. The observed increases in Olsen-P and labile P are consistent with, but do not demonstrate, mechanisms such as metal complexation, competition for sorption sites, microbial mineralization, or altered root growth; these processes were not measured in this experiment. The results of redundancy analysis (**Figure 5**) indicate that the amino acid-enriched phosphate fertilizer contains a relatively high proportion of oxygen-alkyl carbon, carboxyl/amide carbon, and alkyl carbon, which shows a strong positive correlation with wheat yield. In addition, amino acid synergistic substances can be directly absorbed and utilized by plants as nutrients, participate in internal metabolism, and are more conducive to the accumulation of carbohydrates in plants (Wang et al., 2019; Cheng et al., 2025). Furthermore, amino acid synergistic substances can improve the rhizosphere environment and promote root growth and development (Cheng et al., 2021). A more developed and stronger root system allows plants to investigate a greater volume of soil, uptake phosphorus more effectively, and consequently indirectly mitigate the constraints in phosphorus mobility and availability that usually occur because of phosphorus fixation during later growth phases (Li et al., 2024; Long et al., 2024; Raidasari et al., 2025). The earlier soil culture experiment additionally validated that, in comparison to traditional phosphorus fertilizers, using AP can significantly lower soil pH, diminish soil P fixation, boost the levels of Ca_2_-P and Ca_8_-P in the soil, postpone the conversion of Al-P to Fe-P, increase the amount of available P, and facilitate root growth (Cheng et al., 2024). The overall effects enhance the ability of plants to absorb nutrients and improve the efficiency with which phosphate fertilizers are utilized. Additionally, this process is vital for managing the transformation of nutrients within soil and fertilizers, while also markedly fostering plant growth and the development of yield by affecting the activity and variety of soil microorganisms along with their related enzymatic systems (Young et al., 2019; Xiang et al., 2023).

Fertilizers containing phosphorus are commonly utilized in the production of grains; however, a significant amount of the applied phosphorus is lost because of inefficient use. Due to their low mobility and strong capacity for fixation, a large quantity of the phosphorus that is applied becomes immobilized via chemical and microbial activities. As a result, only a minor fraction is taken up by crops during the same growing season, with the remainder remaining bound in soil complexes, which decreases efficiency and raises environmental issues like eutrophication (Chowdhury et al., 2017; Huang et al., 2017; Wei et al., 2024; Peng et al., 2025). Enhanced fertilizer types, application techniques, and management approaches are essential for better PUE (Lukowiak et al., 2016; Hopkins and Hansen, 2019; Cong et al., 2020; Damar et al., 2021; Li et al., 2022; Zou et al., 2022; Gong et al., 2025; Luo et al., 2026). However, seasonal PUE continues to be comparatively low (Dhillon et al., 2017; Xing et al., 2021). Indicators such as Accumulative apparent efficiency of phosphorus, physiological use efficiency of phosphorus, AEP and PFPP are useful for evaluating improvements resulting from fertilizer strategies. In this study, AP significantly improved Accumulative apparent efficiency of phosphorus, physiological use efficiency of phosphorus, AEP and PFPP, Among them, AP0.5 gain is the best. This also confirms that amino acid added urea can increase the absorption and utilization of phosphorus in wheat. Possibly attributed to the existence of amino acids—a natural organic colloid with complex structures that impact phosphorus dynamics (Yang et al., 2019; Volf and Rosolem, 2021; Zhang et al., 2024). The dissociation of hydrogen ions by hydroxyl, carboxyl, and aromatic groups in amino acids activates phosphorus and inhibits its precipitation (Cheng et al., 2024). Additionally, amino acids elevate the quantity of competitive adsorption locations, thereby enhancing phosphorus availability. These findings suggest that phosphorus fertilizers fortified with amino acids can help equilibrate soil phosphorus reservoirs, increase yield and phosphorus efficiency, and promote environmentally sustainable agricultural practices.

### 4.3 Correlations between Olsen-P and P fractions

Olsen-P quantifies the crop-available phosphorus pool (Johnson et al., 2006; Fu et al., 2021; Cheng et al., 2024). Correlation analysis between phosphorus fractions and Olsen-P is commonly used to indicate the relative bioavailability of the former (Shen et al., 2019; Wu et al., 2020; Steinfurth et al., 2021; Wang et al., 2021; Fang et al., 2026). In this study, most soil phosphorus fractions were significantly correlated with Olsen-P, with H_2_O-P, NaHCO_3_-P_i_, and NaOH-P_i_ showing relatively high correlation coefficients. Furthermore, partial least squares regression (PLSR) analysis indicated that these three phosphorus fractions had the highest variable importance in projection. These results suggest that these soil phosphorus forms have relatively high bioavailability and contribute significantly to Olsen-P. However, correlation coefficients and variable importance in projection may not be sufficient to characterize the direct or indirect effects of phosphorus fractions on Olsen-P (Chen et al., 2022b). Path analysis has been proven to be more effective for assessing the source-sink relationships of bioavailability and understanding the transformation processes of soil phosphorus among different phosphorus fractions (Chen et al., 2022b; Liao et al., 2022; Zhu et al., 2022). In this study, the results of path analysis indicated that H_2_O-P, NaHCO_3_-P_i_, and NaOH-P_i_ all had direct effects on Olsen-P, while NaHCO_3_-Po and NaOH-Po may contribute indirectly to Olsen-P through their mineralization to NaHCO_3_-Pi and NaOH-P_i_, respectively. This may be because the application of amino acid-enhanced phosphate fertilizer affects the “crop-fertilizer-microorganism” system, leading to the conversion of more organic phosphorus into inorganic phosphorus to maintain the soil Olsen-P level. This can be explained by the “nitrogen mineralization theory” of soil organic matter priming effect (Craine et al., 2005). The input of exogenous carbon promotes microbial growth, increases their demand for inorganic nitrogen, and stimulates their mineralization and decomposition of soil organic matter to obtain inorganic nitrogen. This process is also accompanied by the conversion of organic phosphorus to inorganic forms, thereby increasing the soil inorganic phosphorus pool (Lu et al., 2022).

## 5 Conclusions

Simply reducing the application rate of P fertilizer does not guarantee high yields. However, adopting amino acid enhanced P fertilizers offers a promising strategy for managing P more efficiently maximizing yield and PUE while minimizing environmental impacts. This study found that the application of amino acid-enhanced phosphate fertilizer could promote the development of effective panicles, improve the activation and absorption processes of soil phosphorus, and significantly increase the grain yield and phosphorus absorption efficiency of wheat. In particular, the effect of AP0.5 was the most significant. From the analysis of soil phosphorus dynamics, it can be seen that the amino acid-enhanced phosphate fertilizer can increase the concentration and proportion of active phosphorus components, and at the same time reduce the content of stable phosphorus components. This change promotes the activation of phosphorus and significantly increases the level of available phosphorus in the soil. Furthermore, path analysis between different phosphorus fractions and Olsen-P indicated that H_2_O-P, NaHCO_3_-P_i_, and NaOH-P_i_ had the greatest direct effects on Olsen-P, while NaHCO_3_-P_o_ and NaOH-P_o_ may be converted into Olsen-P via NaHCO_3_-P_i_ and NaOH-P_i_. This result suggests that amino acid-enriched phosphate fertilizer enhances the substitution for phosphorus fertilizers, leading to greater conversion of organic phosphorus into inorganic phosphorus to maintain soil Olsen-P levels. Despite these positive results, the complexity of phosphorus cycling within the annual soil phosphorus pool in rice-wheat rotation systems warrants further investigation.

## Data Availability

The raw data supporting the conclusions of this article will be made available by the authors, without undue reservation.
