# Correction: Amino acid enhanced phosphorus fertilizers enhance wheat yield and efficiency by optimizing soil phosphorus pools in wheat fields for soil phosphorus risk management

**DOI:** 10.3389/fpls.2026.1983416

**Published:** 2026-09-14

**Authors:** Lin Cheng, Linchuan Zhan, Zongya Wang, Ximei Cao, Tingting Xu, Hongyan Wu

**Affiliations:** 1 Department of Hydraulic Engineering, Wanjiang University of Technology, Maanshan, Anhui, China; 2 Anhui Provincial Key Laboratory of Nutrient Cycling and Arable Land Conservation, Soil and Fertilizer Institute, Anhui Academy of Agricultural Sciences, Hefei, Anhui, China

**Keywords:** amino acid enhanced phosphorus fertilizer, phosphorus fractions, phosphorus use efficiency, soil phosphorus pool, wheat

An incorrect number was provided for the “Natural Science Foundation of Anhui of China”. The correct number is “2508085QC110”.

The original version of this article has been updated.

